# The Ethics of Medical Visiting Again

**Published:** 1889-02

**Authors:** 


### *THE ETHICS OF MEDICAL VISITING AGAIN.*

In a former number of this JOURNAL we took occasion to notice a letter of Dr. D. A. K. Steele, of Chicago, in which he gave some of his impressions of European surgeons. A second letter on a similar subject, published in the same journal, *The Western Medical Reporter*, again attracts our attention, and with it, it is our purpose now to deal.

Dr. Steele, of Chicago, Ulysses-like, has continued his wanderings, but unlike the crafty "Dux Danaorum," does not escape the machinations of the Circe.

Dr. Steele, we suspect, carries his Circe with him. She seems with him too much at home not to be "to the manner born." As Socrates had his "Daimon," so has Dr. Steele, of Chicago, his Circe. Here the comparison must end. Dr. Steele neither teaches as Socrates, nor laughs as Democritus. Neither, like Diogenes, does he sit in a tub, but like him he sneers and scorns. Here, too, the comparison must end, for Diogenes, we are told, *sat* in his tub, content to sneer at what came along. He neither left home to sneer, nor published his diatribes upon mankind in "The Sinope Slanderer." Once, it is currently reported, the philosopher lighted his lamp in broad daylight, and went in search of a man. Dr. Steele, of Chicago, apparently, at cross purposes with his pseudo-prototype, in a fit of despair, girded up his loins and went into a far country, blinded by the glare of Chicago, to seek a surgeon, or rather to discover whether surgery, as a fine art, had any existence—except as a lost art—outside of Chicago. Whether Diogenes in his search came upon a man, we are left to conjecture. Not so with Dr. Steele. For him all the world—but Chicago—-is one great Nazareth. And "can any good come out of Nazareth"? Like the monks of old, Dr. Steele is lost in self-contemplation. Like St. Simon Stylites, perched upon his pillar, too far from the spheres to hear their music, and too far from the earth to be a part of it, or to have sympathy with it, for him there is but one imposing figure—the perch and its occupant.

Persons, like mirrors, are faulty aids for scientific observations. By the first, one can see only what perchance passes. By the second, he can see only himself and what is behind. A combination of mirror and pedestal is particularly liable to induce self-worship. We speak plainly. We are in earnest, and have no time to waste in "patching fig leaves for the naked truth." With Dr. Steele and his methods, we have no sympathy; nor can any man or men, not blinded by a desire for notoriety, nor puffed up by vain egotism, nor altogether lacking in a respect or appreciation of "the unwritten law of medical comity," or common decency.

With Dr. Steele's former letters, both as regards Mr. Tait and other surgeons, we spoke plainly, but not harshly, hoping that a regard for the good name and good sense of Americans generally, would lead him to desist from further perpetration of boorish incivility upon those good enough to admit him to their table and their work. This he refuses to do. In the November number of the *Western Medical Reporter* he thus harangues upon Prof. Péan:

"To my surprise, I found that Péan was deficient in those qualities that mark the difference between a really great surgeon and a [sic] merely skilful operator. He became nervous, irascible, and profane, and soon had all of his assistants in the same unhappy frame of mind. . . . . The first part of the operation was a brilliant success, . . . . but from the moment of hemorrhage occurring from the stump, its management was a dismal failure."

And this is the criticism of a man personally invited by the operator to his private hospital.

Hospitality, both "the unwritten law of medical comity" and the (written) law of self-defense, must soon be a thing of the past, if such conduct as this goes without condemnation and reproval. Dr. Steele's diatribes should be included in the index expurgatorius, and be burned without reading. Dr. Steele should be suppressed.

